# Optimization of some processing parameters and quality attributes of fried snacks from blends of wheat flour and brewers' spent cassava flour

**DOI:** 10.1002/fsn3.255

**Published:** 2015-07-29

**Authors:** Adebukola T. Omidiran, Olajide P. Sobukola, Ajoke Sanni, Abdul‐Rasaq A. Adebowale, Olusegun A. Obadina, Lateef O. Sanni, Keith Tomlins, Tosch Wolfgang

**Affiliations:** ^1^ Departments of Food Science and TechnologyFederal University of AgricultureAbeokutaNigeria; ^2^ Nutrition and DieteticsFederal University of AgricultureAbeokutaNigeria; ^3^ Natural Resources InstituteUniversity of GreenwichGreenwichU.K; ^4^ SABMiller Plc SurreyU.K

**Keywords:** Brewers' spent cassava flour, color, fried snack, microstructure, optimization, sensory

## Abstract

The effect of some processing parameters (frying temperature [140–160°C], frying time [2–4 min], level of brewers' spent cassava flour (BSCF) [20–40%], and thickness [2–4 mm]) on some quality attributes of wheat‐BSCF fried snack was investigated. Response surface methodology based on Box–Behnken design was used to optimize the effect of process parameters on product quality. Sensory evaluation of the optimized sample to determine its level of acceptability was carried out as well as the comparison with fried snack from 100% wheat flour. Increasing temperature had significant (*P* < 0.05) negative effect on the texture. Based on the desirability (0.771) concept, a frying temperature of 140 °C, frying time of 4 min, 32% level of BSCF, and 2 mm thickness was obtained as the optimized conditions. Sensory analyses showed that the optimized sample was preferred in terms of texture and its oiliness to fried snack prepared from 100% wheat flour, but, the aroma, taste and appearance of the wheat snack were preferred.

## Introduction

Many formulated products are based on wheat flour (among other components) and its popularity is largely determined by the ability of the wheat flour to be processed into different products for example, a snack, which is mainly given by the unique properties of wheat‐flour gluten proteins (Anjum et al. 2007). Snack foods are an integral part of the diet which constitute an important part of many consumers' daily nutrient and calorie intake (Amudha et al. 2002) and are typically produced to be durable, accessible, inexpensive, and easy to eat out of a bag or package without further preparation. Some common ones include biscuit, cake, chin chin among others. ‘Chin chin’ is a fried product made from 100% wheat flour, egg, baking powder, and sugar. It is eaten as a snack by all classes of individuals in Nigeria. Cassava is one of the most important food security crops for approximately 700 million people. Postharvest losses are significant and come in three forms which are physical, economic through discounting or processing into low value products, and from bio‐wastes. To reduce these losses, the role that cassava play in food and income security must be enhanced and new market opportunities for new products and added value products must be generated. In the manufacture of beer, various residues and by‐products are generated. The most common ones are spent grains, spent hops, and surplus yeast, which are generated from the main raw materials (Mussatto 2009). The use of high quality cassava flour in brewing generates by‐products called brewers' spent cassava mash which could be useful for food and industrial purposes. Brewers' spent cassava when converted to flour can be a rich source of fiber and protein as well as a functional marketable material. In fact, brewer spent grains obtained from brewing process using cereals has sparked interest in its usage as an adjunct in human food (Mussatto et al. 2006; Sobukola et al. 2012) especially due to an increase in the dietary fiber contents of foods. Spent cassava flour may also be a low‐cost ingredient that can be used for snack production. Frying is an established process of food preparation worldwide. It is a simultaneous heat and mass transfer process where moisture leaves the food in the form of vapor bubbles, while oil is absorbed simultaneously. During the frying process of the snack, the physical, chemical, and sensory characteristics were modified. Various researches have been carried out on the use of brewers' spent grains, by‐products from the use of barley and other grains for food and feed purposes (Awoyale et al. 2011; Sobukola et al. 2012). However, there is no information on the utilization of brewers spent cassava flour. Hence, the objective of this study is to evaluate some quality parameters of fried snacks produced from blends of wheat and brewers spent cassava flour.

## Materials and Methods

The Brewers' spent cassava was obtained from SABMiller, U.K. Wheat flour, vegetable oil, sugar, baking powder, and butter were bought at a supermarket in Abeokuta, Ogun State, Nigeria.

### Preparation of wheat‐brewers spent cassava flour

The brewers' spent cassava was dried in a cabinet dryer at 70°C for 16 h to 7.78% moisture content; milled and sieved using 250 micron to obtain brewers' spent cassava flour (BSCF).Wheat flour and BSCF were weighed and mixed in the ratios as follows: 80:20%, 70:30%, and 60:40%. The various mixes were thoroughly blended and packed in low density polyethylene.

### Product preparation

Dough sample was prepared using the method described by Gazmuri and Bouchon (2009). The dry ingredient proportion was modified to ensure that they all contained the specified amount of water added depending on the initial water content of the ingredients. To every 100 g of flour, 2 g of baking powder, 10 g of butter, and 20 g of sugar were added. The ingredients were mixed; distilled water was added to the dry mixture blend until it reached 40% water content (wb) to form the dough. Half of the water was heated at 100°C and added while mixing at room temperature. After mixing for 2 min, the rest of the water was added. The dough was sheeted to get a final thickness of 2–4 mm which was then cut into cubes and fried.

### Frying experiment

The dough (cubes) were placed inside the frying basket and covered with a grid to prevent them from floating. Frying was carried out in an electrically heated deep fryer (Bush Domestic FCO300, U.K.) containing 3 L of vegetable oil which was preheated to the test temperature for 1 h prior to frying. The product was fried by immersing the product in a wire basket in the oil for 2, 3, or 4 min for the 2 mm, 3 mm, and 4 mm thicknesses, at frying temperature of 140, 150, or 160°C accordingly. After frying, the samples were removed from the fryer and held on a stainless steel grid for 10 min to allow excess oil to drain from the fried products.

### Optimization procedure

A four factor experimental set up was used with frying temperature (*X*_1_), frying time (*X*_2_), BSCF: wheat flour levels (*X*_3_) and sample thickness (*X*_4_) as the independent factors at three levels each as shown in Table 1. The data obtained was analyzed by response surface methodology (RSM) based on Box–Behnken design (Table 2) to optimize process variables. Twenty‐nine combinations including four replicates of the center point was performed in random order according to the design.

**Table 1:** Coded values of the independent variables

Variables	Codes		
	−1	0	+1
Frying temperature (^°^C)	140	150	160
Frying time (min)	2	3	4
Level of BSCF (%)	20	30	40
Thickness (mm)	2	3	4

**Table 2:** Experimental runs showing different combinations of the independent variables

Experimental runs	*X*_1_	*X*_2_	*X*_3_	*X*_4_
_1	150	4	20	3
2	140	3	40	3
3	140	3	30	2
4	150	2	30	4
5	150	3	30	3
6	150	2	40	3
7	150	3	30	3
8	160	4	30	3
9	160	3	30	4
10	150	3	40	4
11	150	3	40	2
12	150	3	30	3
13	150	2	20	3
14	150	3	30	3
15	150	4	30	4
16	150	4	30	2
17	150	4	40	3
18	150	3	20	2
19	140	3	20	3
20	140	2	30	3
21	140	4	30	3
22	140	3	30	4
23	160	2	30	3
24	150	2	30	2
25	160	3	30	2
26	150	3	30	3
27	150	3	20	4
28	160	3	40	3
29	160	3	20	3

Where *X*_1_ = Temperature, *X*_2_ = Frying time, *X*_3_ = Level of BSCF, and *X*_4 _= Thickness.

### Proximate composition

The composite flour (WF/BSCF) and fried snacks from it were analyzed for moisture, ash, and oil according to AOAC (2003). Protein was determined using Kjeldahl method (AACC, 46‐12.01). The carbohydrate content was obtained by difference.

### Color measurement of fried snacks

Color measurement was done using the technique explained by Papadakis et al. *(* 2000). This was carried out by setting up a lightning system, using a high‐resolution camera to capture images and Photoshop software to obtain color parameters. The image acquisition system consists of a color digital camera, Samsung HD 5X model which was used alongside a large box impervious to light with internal black surfaces. L, a, b coordinates was obtained using Adobe Photoshop 6.0 software, which was normalized to L*, a*, b* coordinates, according to equations (1), (2), (3) (Yam and Papadakis 2004).

(1) $$L^{⋆}=\frac{L}{255}\times 100$$

(2) $$a^{⋆}=a\times \frac{240}{255}-120$$

(3) $$b^{⋆}=b\times \frac{240}{255}-120$$

The color difference between the raw (*L*_o_*, *a*_o_*, *b*_o_*) and fried (L*, a*, b*) snack was determined by taking the Euclidean distance between them, according to Mariscal and Bouchon (2008) shown in equation (4):

(4) $$\Delta E{\;}^{⋆}={\left({\left(L_{o}^{⋆}-L^{⋆}\right)}^{2}+{\left(a_{o}^{⋆}-a^{⋆}\right)}^{2}+{\left(b_{o}^{⋆}-b^{⋆}\right)}^{2}\right)}^{\frac{1}{2}}$$

### Expansion analysis of fried snacks

Expansion was determined using a micrometer screw gage and was defined as the maximum height developed during frying (Gazmuri and Bouchon 2009). Reported values represent the mean of six measurements for each frying condition.

### Texture measurement of fried snacks

Hardness of fried snacks was measured using the Texture analyser as described by Da Silva and Moreira (2008) using a three‐point bending test where the sample is supported at two parallel edges and the load is applied centrally. The force (*N*) at the fracture point (highest value in the plot) was used as the resistance to breakage. The mean of three measurements for each frying condition is reported.

### Sensory analysis of fried snacks

#### Acceptance test

The acceptance test was determined using the method described by Ihekoronye and Ngoddy (1985). Fifty consumer panellists made up of students of Federal University of Agriculture, Abeokuta, Ogun State, Nigeria evaluated the appearance, color, texture, oiliness, taste, and overall acceptability of fried snack prepared using the optimized frying conditions on a seven‐point hedonic scale ranking seven for like extremely and one for dislike extremely. The average and mean values of scores for each of attributes was computed and analyzed statistically.

#### Preference test

A preference test was conducted to evaluate the sensory properties of fried snack from 100% wheat flour and optimized conditions. Thirty panelists made up of students of Federal University of Agriculture, Abeokuta, Ogun State, Nigeria were asked to compare each coded sample on basis of some specified characteristics (taste, aroma, texture, and overall appearance). Responses of the panellists were then analyzed statistically (Da Silva and Moreira 2008).

### Scanning electron microscopy

The fried snacks were superficially defatted by immersing them in petroleum ether 35–60 for 2 h after frying. The samples were then coated with a thin gold layer (20 nm) using a Varian Vacuum Evaporator PS 10E (Evey Engineering's Warehouse, Hoboken, NJ, USA) and analyzed using a variable pressure scanning electron microscope LEO 1420VP (LEO Electron Microscopy Ltd., Cambridge, U.K.) at an acceleration potential of 25 kV. An Oxford 7424 solid‐state detector (Oxford Instruments, Oxford, U.K.) was used to obtain the electron microphotographs (Sobukola et al. 2013).

### Statistical analysis

A second‐order polynomial model for the dependent variables as shown in equation (5) was established to fit the experimental data. An analysis of variance (ANOVA) test was carried out using Design‐Expert Version 6 (Stat‐Ease, Inc., Minneapolis, MN) to determine level of significance at 5% level. The generalized regression model fitted was

(5) $$Y=\beta_{o}+\sum_{i=1}^{4}\beta_{i}X_{i}+\sum_{i=1}^{4}\beta_{ii}X_{i}^{2}+\sum \sum_{i/gtj=1}{}^{4}\beta_{ij}X_{i}X_{j}+\epsilon$$

where *Y* is the response; *β*o is a constant; while *β*_*i*_, *β*_*ii*_ and *β*_*iii*_ are linear, quadratic, and interaction coefficients, respectively; and *ε* is error.

## Results and Discussion

### Proximate composition of the flour and fried snacks

Proximate composition of the blends of brewers' spent cassava flour (BSCF) and wheat flour (WF) ranged as follows; 7.78–13.16% moisture, 13.14–16.01% protein, 2.50–3.65% ash, 67.74–69.11% carbohydrate, and 2.75–4.61% fat as presented in Table 3. The regression coefficients of the fried snacks vary between 0.42 and 0.76 as shown in Table 7. There were significant (*P* < 0.05) differences in the proximate composition of the blends. The higher protein content of the whole BSCF flour could be attributed to the addition of enzymes which are proteins to the cassava flour during brewing process to aid the conversion of starch into sugars. The proximate composition of the fried snack from BSCF and wheat blends have the protein, moisture, ash, oil, carbohydrate content, and total dietary fiber, values ranging between 7.97–9.22%, 3–12.5%, 2–3%, 12.82–46.14%, 33.96–67.24%, and 3.43–3.87, respectively, as shown in Table 4. The shelf life of the fried products is mostly determined by the moisture content after frying and the values observed in this work suggests that the frying process reduces the final moisture contents of some of the fried snack products to a level that might be shelf stable. Ashworth and Draper (1992) reported that high‐moisture products (>12%) usually have shorter shelf stability compared with low‐moisture products (<12%). The lower the initial moisture content of a product, the better the storage stability of the product (Akubo 1997). The moisture level of the snacks decreased during frying as water vaporized, oil penetrated into the food. Ash content is similar in all products at 2–3%. The ash content of the fried snacks was noted to decrease significantly compared with the level in the BSCF itself. This could be attributed to the high level of wheat flour used (60, 70, and 80%) in all cases as these were sufficient enough to reduce its level in all samples. Oil content of the snacks reduced with increased frying time. The results may be explained by the formation of a crust, which acts as a barrier to reduce the oil uptake. The crust formation prevents the inside water from escaping to the outside and consequently preventing further oil uptake. Oil absorption is affected by the porosity of the product. Porosity increases during frying and longer frying times resulted in more uniform pore size distribution (Kawas and Moreira 2001). There were no significant (*P* < 0.05) effects by frying temperature, frying time, level of BSCF, and thickness on carbohydrate content.

**Table 3:** Proximate composition of Brewers' spent cassava – wheat flour blends

	A	B	C	D	E
Protein (%)	16.01^d ^± 0.92	14.09^c ^± 0.14	14.0^c ^± 0.18	13.7^b ^± 0.50	13.14^a ^± 0.14
Fat (%)	4.61^d ^± 0.02	2.94^b ^± 0.05	2.75^a ^± 0.05	3.02^bc ^± 0.02	3.06^c ^± 0.04
Ash (%)	2.50^a ^± 0.08	2.88^a ^± 0.18	3.65^b ^± 0.26	3.58^b ^± 0.18	2.88^a ^± 0.07
Moisture (%)	7.78^a ^± 0.13	10.83^b ^± 0.07	11.87^c ^± 0.11	11.98^c ^± 0.18	13.16^d ^± 0.23
Carbohydrate (%)	69.11^b ^± 0.14	69.27^b ^± 0.04	67.74^a ^± 0.02	67.73^a ^± 0.39	67.77^a^ ± 0.20

Mean values followed by different superscript within the same row are significantly different (*P* < 0.05). Values are means of duplicates; A = 100% BSCF, B = 60:40 (W:BSCF), C = 70:30 (W:BSCF), D = 80:20 (W:BSCF), and E = 100WF.

**Table 4:** Response surface analysis results of proximate composition of fried snack for the experimental runs

Runs	Protein (%)	Moisture (%)	Ash (%)	Oil content (%)	Carbohydrate (%)	Total dietary fiber (%)
1	8.44	4.50	3.00	19.06	61.27	3.73
2	7.97	8.20	2.50	25.16	52.60	3.57
3	8.54	4.70	2.50	22.12	58.60	3.54
4	8.63	12.50	2.00	22.43	50.68	3.76
5	8.34	6.90	2.00	20.39	58.81	3.56
6	8.40	6.60	2.00	46.14	33.14	3.72
7	8.69	9.70	3.00	44.65	30.35	3.61
8	8.65	9.50	2.50	19.32	56.27	3.76
9	8.68	8.80	2.00	22.85	53.80	3.87
10	8.25	11.10	3.00	24.40	49.48	3.77
11	8.39	3.90	2.00	25.45	56.62	3.64
12	8.13	6.60	2.00	19.33	60.51	3.43
13	8.79	8.20	2.00	17.89	59.47	3.65
14	8.82	7.90	2.00	19.26	58.46	3.56
15	8.87	6.00	3.00	19.24	59.37	3.52
16	8.51	3.00	2.50	21.78	60.38	3.83
17	8.24	4.10	2.00	23.81	58.11	3.74
18	8.61	4.10	2.00	18.29	63.25	3.75
19	8.43	9.20	2.00	27.34	49.39	3.64
20	8.79	9.60	2.00	20.69	55.20	3.72
21	9.22	5.30	2.00	16.94	62.78	3.76
22	8.84	8.60	2.50	12.82	63.45	3.79
23	8.74	9.50	2.00	19.77	56.38	3.61
24	7.99	7.30	2.50	23.94	54.51	3.76
25	8.29	4.90	2.00	20.20	61.07	3.54
26	8.76	7.40	2.00	20.12	58.29	3.43
27	8.21	7.60	2.50	18.53	59.53	3.63
28	8.46	4.80	2.00	23.21	57.72	3.81
29	8.80	8.00	2.00	21.00	56.57	3.63

Values reported are means of duplicates.

### Expansion and color of fried snacks

Table 5 shows the results of expansion and color parameters (texture, lightness, redness, yellowness, and change in color) of the fried snacks which ranged from 3.85 to 6.98 mm, 86.7 to 95.64, −1.48 to 4.03, 14.63 to 20.92, and 2.90 to 91.20, respectively. Expansion of the product was reported as the maximum height attained under different experimental conditions. Expansion decreased due to the fact that BSCF does not contain gluten that will support maximum expansion. Expansion was significantly (*P* < 0.05) affected by the level of BSCF, as the level of BSCF increased, it reduced expansion. Gazmuri and Bouchon (2009) and Sobukola et al. (2012) while working on fabricated matrices from wheat starch and vital gluten reported that products containing high amount of gluten and water tend to expand during frying with the gluten content of the matrix developing an elastic structure that traps water vapor producing an expanded product.

**Table 5:** Response surface analysis results of color parameters, expansion, and texture of fried snack for the experimental runs

Runs	Expansion (mm)	Texture (*N*)	Lightness	Redness	Yellowness	Change in color
1	6.26	45.90	94.61	−1.17	16.10	41.62
2	3.85	13.60	93.15	−0.37	14.63	14.99
3	5.29	32.80	92.94	1.07	19.05	10.53
4	5.83	26.70	90.72	2.15	20.53	21.88
5	5.44	29.00	93.07	0.77	17.04	14.77
6	5.89	21.05	87.11	4.03	20.92	23.47
7	5.77	21.40	92.82	1.29	19.87	10.51
8	5.03	34.60	92.12	1.06	18.81	13.64
9	6.98	34.70	92.76	0.64	18.73	11.15
10	4.94	26.40	92.05	0.76	19.11	2.90
11	5.91	18.00	86.70	3.40	20.82	21.84
12	5.97	38.15	94.12	−0.65	16.04	13.67
13	5.79	42.30	94.05	−0.19	19.24	75.26
14	6.13	36.80	95.64	−0.70	16.27	11.11
15	6.90	43.50	93.70	0.63	17.25	12.09
16	6.01	13.20	93.46	0.37	17.30	11.78
17	5.98	23.60	91.66	1.47	19.67	4.28
18	5.89	19.10	92.08	1.20	19.25	91.27
19	5.88	60.10	94.89	−0.49	17.32	51.54
20	5.96	28.30	93.41	0.45	18.34	9.02
21	6.16	31.40	91.14	1.47	20.10	17.40
22	6.07	67.30	94.83	−0.17	16.43	11.73
23	4.88	17.50	94.15	−0.29	18.95	4.63
24	6.10	18.00	93.22	0.25	20.48	6.08
25	6.16	17.80	94.55	0.37	17.10	10.52
26	6.16	18.90	92.34	0.48	16.24	30.81
27	6.43	40.20	95.05	−0.88	18.51	61.71
28	5.10	23.20	92.55	0.51	18.62	3.36
29	6.67	30.10	93.89	–1.48	17.65	58.22

Values are means of duplicates.

### Texture of fried snacks

One important quality parameter of desirable textural characteristic of fried foods is crispness because it signifies freshness and high quality. Breaking force reduces if the fried snack becomes crispier and this could be made possible by increasing the frying time and temperature. This is in agreement with Rossell (2001) who reported that at higher frying temperature and time, crust formation is enhanced. At higher frying temperature and time, texture also reduced with increased level of BSCF but increased with thickness.

### Color measurement of fried snacks

The changes in the color of fried products are as a result of the Maillard reaction that depends on the content of reducing sugars and amino acids at the surface, as well as the temperature and frying time as reported by Marquez and Anon (1986). Color is considered as one of the most important quality parameters of deep fat fried snacks. As the frying temperature increased, the lightness parameter of the fried product decreased, whereas the redness and yellowness parameters increased for the same frying time (Krokida and Oreopoulou 2000; Moyano et al. 2002). These results were consistent with this study. Lightness value of fried snack decreased with increase in frying temperature, frying time, and level of BSCF, while redness and yellowness values increased. The addition of wheat flour could be said to cause an increase in the amount of amino acid in the flour blend used for the fried snacks allowing the Maillard browning reaction to easily occur, with increase in level of BSCF resulting in the decrease in lightness value, but increased redness and yellowness values. This was similar to the report of Jirawan et al. (2009). The increase in redness and yellowness values could be attributed to the color of the BSCF which is almost light yellow. At increasing thickness, lightness increased and this could be attributed to increasing quantity of the dough that was fried while redness and yellowness decreased. The lightness and redness was observed to have a positive significant (*P* < 0.05) effect on the level of BSCF but was not significantly affected by frying time, frying temperature, and thickness. Furthermore, the yellowness of the fried snacks was significantly (*P* < 0.05) affected by the interaction between frying time.

**Figure 1:**
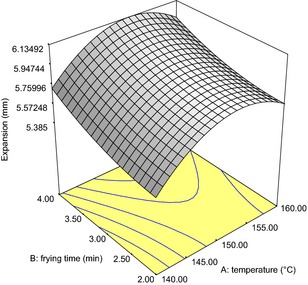
Response surface plot showing effect of independent variables on the expansion (mm) of fried snacks from Wheat‐Brewers' Spent Cassava Flour.

**Figure 2:**
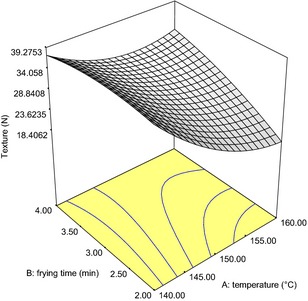
Response surface plot showing effect of independent variables on the texture (*N*) of fried snacks from Wheat‐Brewers' Spent Cassava Flour.

**Figure 3:**
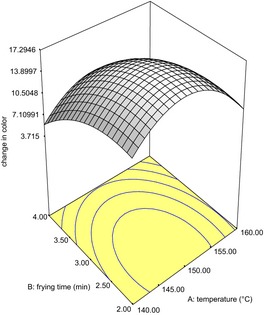
Response surface plot showing effect of independent variables on change in color of fried snacks from Wheat‐Brewers' Spent Cassava Flour.

The regression coefficients of the quality parameters of the fried snack are as shown in Tables 6 and 7 while Figures 1, 2, 3 are the response surface plots for the expansion, texture and change in color, respectively.

**Table 6:** Regression coefficients of the response surface models and statistical results of the color parameters, expansion, and texture of the fried snacks

Coefficients	Expansion (mm)	Texture (*N*)	Lightness	Redness	Yellowness	Change in color
*B*_o_	5.89	28.85	93.6	0.24	17.09	16.17
*X*_1_	0.13	−6.30*	−0.03	−0.1	0.33	−1.14
*X*_2_	0.16	3.2	0.34	−0.21	−0.77	−3.29
*X*_3_	−0.44*	−9.32*	1.78*	1.07*	0.48	−25.73*
*X*_4_	0.15	9.99*	0.51	−0.29	−0.29	−2.55
*X*_1_^2^	−0.27	4.11	0.55	−0.39	−0.12	−5.62
*X*_2_^2^	0.07	−1.56	−0.83	0.54	1.38*	−2.56
*X*_3_^2^	−0.2	0.62	−1.02	0.11	0.65	23.60*
*X*_4_^2^	0.29	−0.1	−0.49	0.49	0.99	1.48
*X*_12_	−0.01	3.5	0.06	0.08	−0.48	0.16
*X*_13_	0.11	9.90*	0.1	0.47	0.92	−4.58
*X*_14_	0.01	−4.4	−0.92	0.38	1.06	−0.14
*X*_23_	−0.09	−0.26	1	−0.4	0.47	3.61
*X*_24_	0.29	5.4	0.69	−0.41	−0.03	−3.87
*X*_34_	−0.38	−3.18	0.6	−0.14	−0.24	2.65
*R* ^2^	0.49	0.76	0.59	0.55	0.53	0.873
PRESS	32.59	5315.3	245.32	95.42	164.32	9638.83
*P*	0.53	0.02	0.25	0.25	0.42	0.0005

*Significant values at 5% level; *B*_o_ is intercept, *X*_1_ − *X*_14_ are regression coefficients.

**Table 7:** Regression coefficients of the response surface models and statistical results of the proximate composition of the fried snacks

Coefficients	Protein	Moisture	Ash	Oil content	Carbohydrate	Total dietary fiber
*B*_o_	8.55	7.70	2.20	24.75	53.28	3.52
*X*_1_	−0.01	−0.01	−0.08	0.11	−0.02	0.02
*X*_2_	0.05	−1.78*	0.21	−2.56	4.07	0.01
X_3_	−0.13	−0.24	0.00	3.84	−3.48	0.02
X_4_	0.10	2.23*	0.13	−0.96	−1.51	0.02
$X_{1}^{2}$	0.12	0.45	−0.12	−3.13	2.60	0.07
$X_{2}^{2}$	0.10	−0.17	0.07	−0.68	0.57	0.11*
$X_{3}^{2}$	−0.18	−0.89	0.00	1.73	−0.73	0.08
$X_{4}^{2}$	−0.08	−0.62	0.19	−3.05	3.47	0.09*
*X*_12_	−0.13	1.08	0.13	0.83	−1.92	0.03
*X*_13_	0.03	−0.55	−0.13	1.10	−0.52	0.06
*X*_14_	0.02	0.00	0.00	2.99	−3.03	0.02
*X*_23_	0.04	0.30	−0.25	−5.88	5.79	−0.02
*X*_24_	−0.07	−0.55	0.25	−0.26	0.71	−0.08
*X*_34_	0.07	0.93	0.13	−0.32	−0.86	0.06
*R* ^2^	0.42	0.76	0.50	0.44	0.42	0.57
PRESS	6.32	189.67	7.61	2262.59	2664.63	0.82
*P*	0.72	0.02	0.50	0.66	0.72	0.30

*Significant values at 5% level; Bo is intercept, X1 – X14 are regression coefficients where X1, X2, X3 and X4 are frying temperature, frying time, level of BSCF and thickness, respectively BSCF‐ Brewers' Spent high quality Cassava Flour

### Optimization of process variables

Expansion, protein content, carbohydrate content, total dietary fiber, and yellowness were maximized (6.98, 9.22, 67.24, 3.43, and 20.92, respectively, while texture [13.2], ash content [2.0], oil content [12.82], lightness [86.7], redness [−1.48], and change in color [2.90] were minimized). Frying temperature of 140.11°C, frying time of 4 min, level of BSCF of 32.09%, and thickness of 2 mm with a desirability of 0.771 was selected and an optimized sample was prepared under these conditions.

### Sensory evaluation of optimized sample

**Figure 4:**
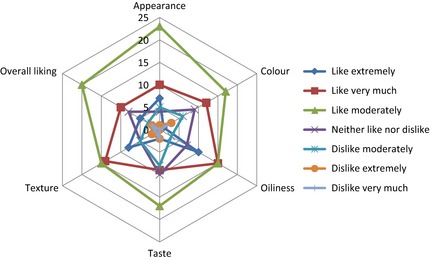
Radar chart showing the level of acceptability of the optimized fried snack based on its attributes.

**Figure 5:**
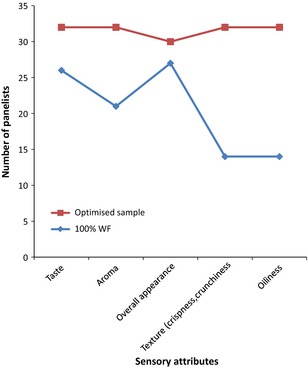
Bar chart showing the results of the preference sensory test.

The result of the sensory evaluation of the fried snacks is presented in Figure 4 which shows the degree of likeness of the optimized sample based on the appearance, color, oiliness, taste, texture, and overall liking. Figure 5 shows the comparison of the optimized sample with 100% wheat flour. The panellists preferred the optimized sample more in texture and greasiness.

### Scanning electron micrographs of fried snacks

**Figure 6:**
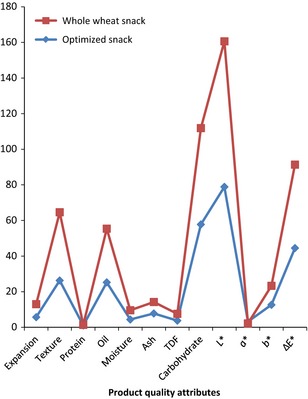
Bar chart showing the results of some quality attributes of the fried snacks.

**Figure 7:**
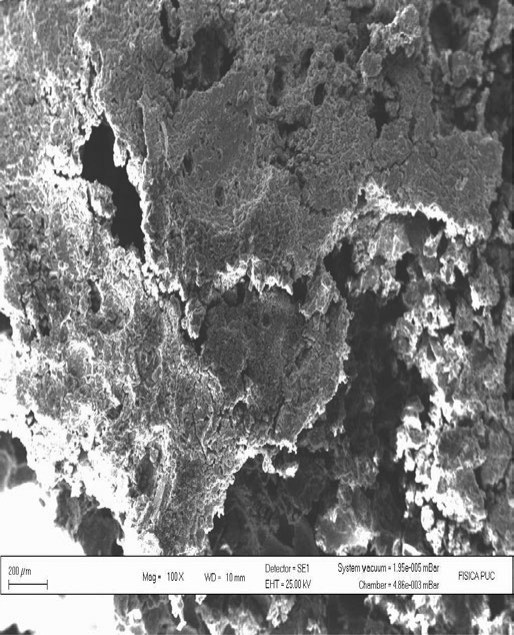
Scanning electron micrographs of fried snacks from 100% wheat flour.

**Figure 8:**
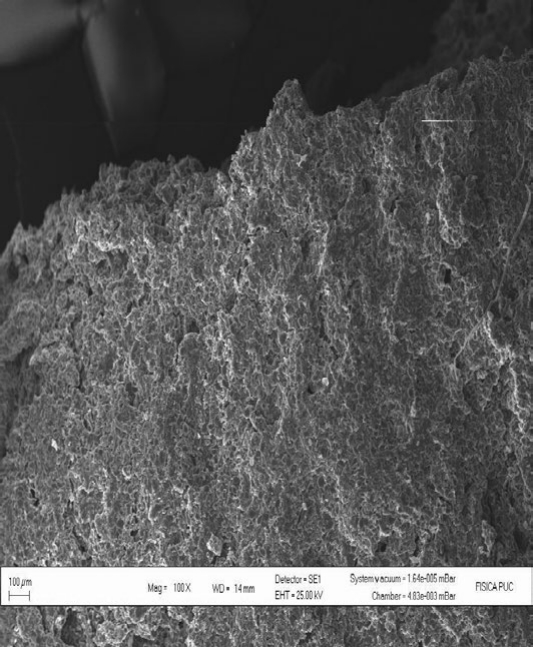
Scanning electron micrographs of fried snacks from wheat plus 32.09% BSCF.

According to Hoseney (1994), wheat starch granules consist of large granules (over 10 *μ*m) and smaller ones (less than 10 *μ*m). The micrograph of fried snack from 100% wheat flour showed the presence of larger air cells formed during frying with less continuity of network which could enhance the migration of oil into the fried snacks, thereby increasing the oil content while the micrograph of the optimized snack had air cells that are smaller in size, more continuity, and less porosity which might help in minimizing oil migration into the fried snack. Though most snacks require a porous texture for their desired sensory properties, the pores should not be large enough to encourage oil migration. Result showed that fried snack from 100% WF had higher oil uptake than the optimized snack as shown in Figure 6 and this is supported by the scanning electron micrographs of the fried snacks (Figs. 7 and 8).

## Conclusion

It can be inferred from this study that an acceptable fried snack can be developed from the inclusion of BSCF to wheat flour. The frying temperature of 140 °C, frying time of 4 min, level of BSCF of 32%, and 2 mm thickness was selected to give maximum values for the various responses with the highest desirability value of 0.771. The optimized fried snack (containing 32% BSCF) had a higher protein content and lower oil uptake than the snack from 100% WF. Also, the optimized fried snack was crispier and requires less breaking force than the snack from 100% WF.

## Conflict of Interest

None declared.
